# Diabetes mellitus awareness among students attending a public high school in Kurdistan Region of Iraq: a cross-sectional study

**DOI:** 10.1097/MS9.0000000000003046

**Published:** 2025-03-03

**Authors:** Ahmed A. Mosa, Hajar Hassan Abdulqadir, Rojeen Chalabi Khalid, Alaa Abdullah Mustafa, Hajar Ahmed Zaki, Iman Ramadhan Yousif, Osama Subhi Yaseen, Steven Esho Dinkha, Haneen Majeed Khamo, Ameen M. Mohammad

**Affiliations:** a College of Medicine, University of Zakho, Kurdistan Region, Iraq; b College of Medicine, University of Duhok, Kurdistan Region, Iraq; c General Directorate of Health-Duhok, Ministry of Health, Kurdistan Region, Iraq; d Department of Internal Medicine, College of Medicine, University of Duhok, Kurdistan Region, Iraq

**Keywords:** awareness, diabetes mellitus, high school students, Iraq, knowledge, Kurdistan Region

## Abstract

**Background and aim:**

Diabetes mellitus (DM) is a chronic metabolic disorder and a significant global concern due to its steadily increasing prevalence. Educating youths and adolescents about such diseases is crucial for promoting healthy lifestyle choices. This study aims to evaluate the knowledge of public high school students about DM and identify areas where their understanding is lacking.

**Materials and methodology:**

In February 2023, a descriptive cross-sectional study was conducted in the Duhok province of the Kurdistan Region in Iraq. The study enrolled 392 students from a public high school, who were surveyed using a self-administered questionnaire. This questionnaire had two sections: the first collected basic demographic information of the participants, while the second included 20 items assessing various aspects of DM knowledge, such as general information, risk factors, signs and symptoms, complications, and prevention.

**Results:**

The mean age of the study participants was 16.4 years (±0.71). Among the participants, 247 (63%) were female, and 190 (48.5%) had a positive family history of DM. Unfortunately, only 53 students (13.5%) had received training programs about the disease, although the vast majority (89.3%) expressed willingness to participate in the prevention programs. For 70.2% of the participants, family and friends were the primary sources of information. The mean knowledge score was 13.9 (±3), with an average correct answer rate of 68.55%. Female students, those with a positive family history, had significantly demonstrated a better overall knowledge score with a mean knowledge score of 14.14 ± 2.96 and 14.17 ± 2.98, respectively. Also, participants using multiple sources for information, and those who had received training programs on DM, demonstrated a better overall knowledge score with a mean score of 14.21 ± 2.90 and 14.34 ± 3.31, respectively. However, it missed the significance.

**Conclusion:**

The study participants exhibited good knowledge and awareness of DM. However, there were notable gaps in their understanding of the disease’s risk factors and complications. Therefore, we recommend incorporating health education programs and lifestyle modification initiatives into the school curriculum to enhance students’ knowledge and address the identified gaps.

## Introduction

**Highlights**

- The average knowledge score among participants was 13.9 ± 3.
- Female students and those with a positive family history of DM exhibited higher knowledge levels.
- Awareness programs about DM are scarce among high school students.
- There is a critical need to integrate health education programs into the school curriculum.

Diabetes mellitus (DM) is a significant global public health issue. This chronic metabolic disorder is marked by elevated blood glucose levels resulting from impaired insulin secretion, insulin resistance, or both. Sustained high blood glucose levels can cause serious and potentially life-threatening complications affecting the heart, kidneys, eyes, and nerves[1]. The prevalence of diabetes is increasing worldwide due to population growth, aging, urbanization, lifestyle changes, physical inactivity, and obesity^[^2,3^]^. According to the International Diabetes Federation, 537 million adults (aged 20–79 years) are currently living with diabetes, and this number is projected to rise to 783 million by 2045[4]. In Iraq, the prevalence of DM is about 2 million and it is estimated that approximately 5000 cases of children and adolescents (aged 0–19 years) are affected by type 1 DM[5]. However, specific data regarding the prevalence of type 2 DM in this age group and young adults are currently lacking.

The prevalence of DM is highest in Middle Eastern countries, and the incidence of type 2 DM is rising among adolescents and youth[6]. Today’s youth are increasingly adopting unhealthy lifestyles, characterized by the excessive consumption of fast food, carbonated drinks, and energy drinks, along with physical inactivity and tobacco smoking. These behaviors elevate their risk of developing DM at a young age[7]. An unhealthy lifestyle, combined with misconceptions and the growing prevalence of the disease, increases the likelihood of individuals being affected^[^8,9^]^. Each person plays a vital role in the prevention and management of health issues. Therefore, it is crucial to disseminate adequate knowledge about the causes, risk factors, signs and symptoms, complications, and prevention of DM to the population, especially young people. This knowledge will foster early adoption of healthy lifestyles, encouraging sustained healthy habits throughout their lives^[^10,11^]^.

Several studies have been conducted, in Iraq and Iraqi Kurdistan, among various population groups to assess their awareness about DM. These studies consistently reported knowledge deficits, particularly among primary school teachers and the general population. Nonetheless, university students demonstrated a better awareness about DM ^[^8,12,13^]^. Although diabetes awareness is vital, no study has examined the awareness of DM among public high school students. This study aimed to bridge this gap by evaluating students’ level of knowledge on DM, including its causes, risk factors, manifestations, complications, and prevention. By identifying knowledge gaps and the factors influencing awareness, this research will aid in developing health education initiatives and awareness programs for students and youth, which will equip students with sufficient information about diabetes and potentially impact its incidence.

## Material and methodology

### Study design and data collection

A descriptive cross-sectional study was carried out among a sample of students in a public high school in Duhok province, Kurdistan Region of Iraq. The data collection took place from 15 February to 26 February 2023. A self-administered questionnaire was distributed among the students comprising various aspects of knowledge about DM as a primary research instrument. A total of 392 high school students were enrolled in the study. To overcome potential language barriers, Kurdish translation was orally provided to students who were not proficient in the English language, ensuring inclusivity and facilitating their participation in the study. The research was designed and carried out in accordance with the Strengthening the Reporting of cohort, cross-sectional, and case-control studies in Surgery criteria[14].

A total of 500 high school students were initially contacted for the purpose of this study. Among those students, 403 students have filled the questionnaire, resulting in a response rate of 80.6%. Due to incomplete filling of the questionnaire, 11 students were excluded. The total number of students enrolled in the final analysis was 392. This sample is sufficient to represent a population of over 50 000 with a confidence interval of 95%, and a margin of error 5%. This was calculated using an online calculator (http://www.raosoft.com/samplesize.html).

### Study tool

This study’s questionnaire was based on the previously validated questionnaires, with some adjustments made by the authors to suit the study participants^[^15,16^]^, with the aim of evaluating high school students’ knowledge about DM. However, before commencing data collection, experts evaluated the final version of the questionnaire for relevance, comprehensiveness, content validity, and cross-cultural adaptation.

The study questionnaire was structured into two distinct sections. The first section covered essential demographic characteristics of the participants, including age, gender, DM status, family history of DM, sources of knowledge about DM, and whether they had received or believed in the necessity of a DM prevention program in schools.

The second section consisted of 20 items to assess the students’ knowledge about DM. This section was further divided into five subcategories. The first category comprised of five questions to measure participants’ general information about DM. The following section assessed students’ awareness of disease risk factors through four relevant questions which included (eating fast food, smoking tobacco, obesity, and physical inactivity). The third category in the knowledge section focused on the students’ familiarity with signs and symptoms associated with the condition. The fourth category delved into the participants’ knowledge of potential complications linked to DM, with three specific questions. The last category of this section inquired about the participants’ understanding of preventive measures related to DM. For this section, students were required to answer “Yes,” “No,” or “I don’t know” to each statement. The correct answer was scored 1, while a score of 0 was given for incorrect or I don’t know. The total score for the knowledge section ranged from 0 to 20.

### Inclusion/exclusion criteria

The eligibility criteria were participants who were actively enrolled in the selected public high school, their age ranged from 15 to 18 years, and who voluntarily provided informed consent to be enrolled in the study. Conversely, individuals who did not provide informed consent, questionnaires with incomplete or missing data, and students not enrolled in the selected public high school or absent during the period of data collection were excluded from the study.

### Ethical approval

The Ethics and Scientific Committee at the University of Zakho, College of Medicine, Kurdistan Region of Iraq provided formal approval for the final version of the survey on 10 February 2023, with reference number (FEB2023/E02). All participants gave written informed consent on the first page of the questionnaire by ticking the box labeled “I agree to participate in this study.” However, parental consent was waived by the Research Ethics Committee.

### Statistical analysis

Statistical analysis was conducted using Microsoft Excel and GraphPad Prism version 9. Excel was employed for data cleaning and coding, after which the data were transferred to GraphPad Prism for further statistical analysis. Categorical variables were analyzed using percentages and frequencies, while numerical variables were assessed in terms of mean, standard deviation, and median. The differences between responses to questions in each knowledge category were analyzed using the Chi-squared test. The relationship between demographic characteristics and the overall knowledge score was examined using a one-tailed independent *t*-test and one-way ANOVA. The *P*-value <0.05 was considered statistically significant.

## Results

### Basic demographic characteristics of participants

In the present study, a total of 392 students attending a public high school in Duhok province aged between 16 and 18 years were recruited for the study. The mean age of the participants was 16.4 years (±0.71 SD). Among the participants, 63% were female while the remaining 37% were male. Only three participants were a known case of DM. Family history emerged as a significant factor, with about 48.5% of students reporting a positive family history of diabetes. The vast majority of the students (86.5%) have never received a training program on the prevention of DM. Nevertheless, 89.3% of participants agreed that these types of programs are essential to elaborate the knowledge about DM. Table 1 shows the basic demographic characteristics of the study participants.

**Table 1:** Basic demographic characteristics of the participants (*n* = 392)

Variables	*n* (%)
Age (years), mean (SD)	16.4 (±0.71)
Gender	
Male	145 (37)
Female	247 (63)
Do you have DM?	
Yes	3 (0.8)
No	389 (99.2)
Family history of DM	
Positive	190 (48.5)
Negative	202 (51.5)
Have you ever received a training program on the prevention of DM in school?	
Yes	53 (13.5)
No	339 (86.5)
Do you think that training programs about DM are necessary?	
Yes	350 (89.3)
No	42 (10.7)

### Sources of knowledge about DM

**Figure 1.**
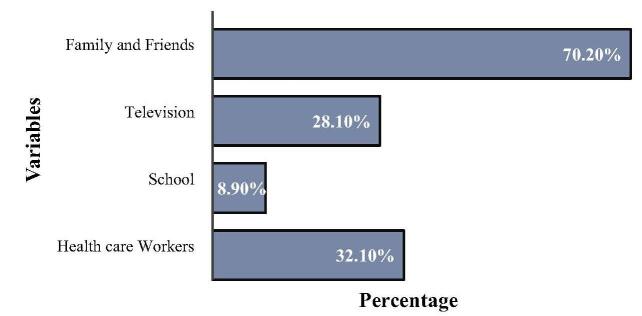
Sources of DM knowledge.

Figure 1 illustrates the common sources of knowledge about DM among the students. Notably, about 70.2% of the participants received information from their family and friends, while approximately one-third obtained their knowledge from health care professionals. In contrast, only 8.9% of the students acquired information from school.

### Participant’s knowledge of DM

Table 2 summarizes the participants’ knowledge about various aspects of DM. In the general knowledge category, 258 students (65.8%) recognized that DM is a condition of high blood sugar, and 294 students (75%) reported that DM is a preventable disease. Conversely, 364 students (92.9%) disagreed with the statement that diabetes only affects older people, and 351 students (89.5%) acknowledged that diabetes cannot be transmitted from person to person. Regarding risk factors, about half of the students were unaware that smoking tobacco is a risk factor for developing DM. However, the majority agreed that obesity could lead to the disease. Additionally, 51.8% and 42.9% of the participants were unaware that eating fast food and physical inactivity, respectively, are risk factors for DM.

**Table 2:** Students’ knowledge about different aspects of DM (*n* = 392)

Variables	Yes *n* (%)	No *n* (%)	I don’t know *n* (%)	*P*-value
**General knowledge about DM**				
1. DM is a condition of high blood sugar level	258 (65.8)	27 (6.9)	107 (27.3)	<0.00001
2. DM is preventable	294 (75)	50 (12.8)	48 (12.2)	
3. DM is curable	70 (17.9)	248 (63.3)	74 (18.9)	
4. DM only affects older people	20 (5.1)	364 (92.9)	8 (2)	
5. DM can be transmitted from person to person	24 (6.1)	351 (89.5)	17 (4.3)	
**Knowledge of risk factors**				
6. Eating fast foods	189 (48.2)	138 (35.2)	65 (16.6)	<0.00001
7. Smoking tobacco	101 (25.8)	209 (53.3)	82 (20.9)	
8. Obesity	314 (80.1)	40 (10.2)	38 (9.7)	
9. Physical inactivity	224 (57.1)	96 (24.5)	72 (18.4)	
**Knowledge of signs and symptoms**				
10. Frequent urination	260 (66.3)	35 (8.9)	97 (24.7)	<0.00001
11. Excessive thirst	309 (78.8)	30 (7.7)	53 (13.5)	
12. Increased feeling of tiredness	241 (61.5)	70 (17.9)	81 (20.7)	
13. Blurred vision	246 (62.8)	57 (14.5)	89 (22.7)	
**Knowledge on complications**				
14. Diabetic patients develop eye problems	237 (60.5)	51 (13)	104 (26.5)	0.0024
15. Diabetic patients develop kidney problems	202 (51.5)	65 (16.6)	125 (31.9)	
16. Diabetic patients’ loss of sensations of limbs	250 (63.8)	61 (15.6)	81 (20.7)	
**Knowledge on prevention**				
17. Healthy diet	368 (93.9)	15 (3.8)	9 (2.3)	0.00011
18. Regular exercise	331 (84.4)	25 (6.4)	36 (9.2)	
19. Weight lose	327 (83.4)	36 (9.2)	29 (7.4)	
20. Regular medical check-ups	334 (85.2)	29 (7.4)	29 (7.4)	

The majority of study participants, numbering 309 (78.8%), recognized excessive thirst as a symptom of DM. Among the students, 260 (66.3%) correctly identified frequent urination as another symptom of diabetes. Approximately, two-thirds of the participants reported that individuals with long-standing diabetes might experience eye and sensation problems, while only half of them acknowledged the potential development of kidney problems. The study findings highlighted the participants’ strong understanding of DM prevention. Over four-fifths of the participants were aware that adopting a healthy diet (93.9%), engaging in regular exercise (84.4%), pursuing weight loss (83.4%), and undergoing regular medical check-ups (85.2%) are effective strategies for preventing and delaying the onset of this disease.

### The score for each knowledge category

Table 3outlines the mean, standard deviation, median, and overall correct answer statistics for each knowledge category. The mean of total knowledge score for this study was 13.9 (±3). The overall average of correct answers in the knowledge section was 68.55%. Within the knowledge section, the prevention category achieved the highest average correct answer rate at 86.7%, followed by general knowledge with an average correct answer rate of 77.3%. Eleven questions had correct answer rates lower than the overall average: two in general knowledge, three in risk factors, three in signs and symptoms, and three in complications.

**Table 3:** Maximum possible score, mean, standard deviation, median, and the average of the correct answers for each knowledge category (*n* = 392)

Knowledge category	Maximum possible score	Mean	Standard deviation	Median	Average correct answer (%)
General knowledge	5	3.86	1.05	4	77.3%
Risk factors	4	2.11	1.1	2	52.8%
Signs and symptoms	4	2.7	1.1	3	67.35%
Complications	3	1.76	0.9	2	58.6%
Prevention	4	3.47	0.87	4	86.7%
Total score	20	13.9	3	14	68.55%

### Determinants of better knowledge score

In our analysis of demographic variables and total knowledge scores, it was observed that female students, individuals with a positive family history of DM, utilizing multiple sources for diabetes knowledge, and those who engaged in a training program exhibited higher overall knowledge scores compared to their counterparts. A significant correlation was identified between overall knowledge and gender, as well as family history, with *P*-values of 0.02 and 0.038, respectively. However, the association between overall knowledge and utilizing multiple sources for information and participation in a training program did not reach statistical significance. Table 4 provides an overview of the factors linked with enhanced knowledge.

**Table 4:** Comparison of the participant’s basic demographic characteristics and overall knowledge level (*n* = 392)

Variables	Mean knowledge score	Standard deviation	*P*-value
Gender			
Male	13.5	±3	0.02
Female	14.14	±2.96	
Family history of DM			
Yes	14.17	±2.98	0.038
No	13.64	±2.98	
Source of DM knowledge			
Health care workers	13.76	±2.99	0.1
School	11.5	±3.74	
Television	13.39	±3.15	
Family and friends	13.94	±2.97	
More than one source	14.21	±2.9	
Have you received a training program			
Yes	14.34	±3.31	0.12
No	13.83	±2.93	

## Discussion

The prevalence of DM is rising, affecting even younger age groups. Adequate health literacy at a young age can enhance the preventive measures taken by individuals resulting in the early detection of diseases, thereby reducing the occurrence of life-threatening conditions. Therefore, we aimed to evaluate the public high school students’ knowledge about DM in the Kurdistan Region of Iraq. The study found that students had a good level of awareness regarding the disease. Participants demonstrated a better understanding of the signs, symptoms, and prevention of diabetes than of its risk factors and complications.

Approximately half of the participants reported a positive family history of diabetes, a value that falls between those reported by two other studies in the region^[^8,17^]^. This discrepancy can be attributed to the lack of an official study accurately documenting the prevalence of the disease and positive family history. The present study highlighted that our educational system rarely focuses on health literacy, with only 13.5% of students reporting that they have attended a DM awareness program in school. This finding aligns with a study conducted in Bangladesh, where only 11% of students had attended educational classes on diabetes[18]. This underscores the urgent need for educational reform to incorporate a special class on health literacy into the curriculum, which would enhance students’ awareness of diseases and subsequently reduce the disease burden on the government.

About two-thirds of our participants reported obtaining information from family and friends, while about one-third cited health care workers as their information source. In contrast, a study in Oman found that social media was the most utilized source of information, followed by health care workers[19]. Relying on non-reliable and nonprofessional sources can lead to the spread of misconceptions and misunderstandings within the community. This issue can be addressed by creating specialized platforms, programs, and groups managed by experts in the field.

In the present study, 65.8% and 75% of respondents correctly defined diabetes as a condition characterized by high blood sugar levels and recognized that the disease can be prevented, respectively. These findings are consistent with a study conducted in the United Arab Emirates[20]. About three-fifths of the participants were aware that diabetes is a chronic disease that can be managed but not cured, although a higher percentage of adolescents in Kuwait were aware of this fact[21]. In our study, the average of overall correct answers in the general knowledge category was 77.3%, higher than the 71% reported in the Kuwaiti study[21].

Eating fast food, smoking tobacco, obesity, and physical inactivity are known to significantly impact human health, contributing to the development of diabetes and increasing morbidity and mortality associated with the disease[22]. Surprisingly, three-fourths of the participants did not know that smoking tobacco is a risk factor for developing diabetes, a figure similar to that found in the Western Region of Saudi Arabia[23]. Only about half of the participants recognized that consuming fast food predisposes individuals to the disease. Unfortunately, our students showed poor knowledge regarding disease risk factors, contrasting with a study conducted among university students in Uganda[15]. This finding highlights the areas where students’ knowledge is lacking, facilitating the effective design of health literacy programs.

Early recognition of symptoms can aid in the early detection and management of diabetes, preventing severe complications. Our results indicated that a relatively high percentage of students were aware of common diabetes symptoms: 66.3% for frequent urination, 78.8% for excessive thirst, 61.5% for increased tiredness, and 62.8% for blurred vision. These results are similar to those found in the research conducted in Tabuk, Saudi Arabia[24].

The present study reveals a low level of knowledge regarding diabetes complications among students. Nearly half of the students did not recognize that diabetic patients can develop kidney problems, which aligns with findings from a study conducted in Ghana[25]. The average percentage of correct answers in the complications category was 58.6%, which is consistent with a study in Muscat, Oman, where 47% of students were unaware of any complications[19]. Regarding prevention, the vast majority of students demonstrated good knowledge of preventive measures. These findings are consistent with a study among Saudi Arabian adolescents and are higher than those from a study in Southern Nigeria^[^16,26^]^.

In our study, students exhibited low knowledge regarding disease risk factors and complications. The average percentage of correct answers in the knowledge section was 68.55%, which is higher than the 63.2% found in a study conducted in Kuwait[21]. Demographic variables such as gender, family history, source of information, and participation in training programs were all associated with better knowledge and understanding of DM among our study participants. We found that females significantly exhibited better awareness of the disease than males (*P*-value = 0.02), consistent with a previous study among university students in Saudi Arabia[27]. However, a study in Nepal among school students found that male students had a slightly higher mean of correct answers compared to females[28]. Studies in Saudi Arabia, Nepal, and Nigeria have reported that students with a positive family history of DM have a better understanding of the disease, a finding that was also significantly reflected in our study (*P*-value = 0.038)^[^27-29^]^.

### Strengths and limitations

To our knowledge, this is the first study in Iraq to evaluate the knowledge and awareness of public high school students about diabetes. However, the study has several limitations. First, a convenience sampling method was employed, and the sample was taken from students at a single public high school in the Duhok governorate, Iraqi Kurdistan, limiting the generalizability of the findings. Nonetheless, the study will be crucial in shaping the future research on diabetes awareness in the region. Second, the questionnaire only included a knowledge section, whereas it is important also to assess students’ attitudes and practices regarding diabetes. Third, the study design was cross-sectional in nature so it cannot establish causality. Finally, the study includes a limited number of covariates, which restricts the ability to perform more complex multivariate analyses. This limitation may impact the robustness of the findings.

### Conclusions and recommendations

The study participants demonstrated a good knowledge and awareness of DM. However, there were significant gaps in their understanding of disease risk factors and complications. The research found that females, those with a positive family history, used multiple information sources, and who had received training programs possessed better knowledge compared to others. To enhance the students’ awareness and address the knowledge gaps identified in some areas of knowledge, integrating health educational programs and lifestyle modification initiatives into the school curriculum is essential. Such inclusion will encourage students to adopt healthy dietary habits and lifestyles from an early age. A study conducted in India showed a significant improvement in students’ knowledge and healthy habits following the implementation of curriculum-based educational programs[30]. In the modern world, social media and the internet are crucial for disseminating information. Therefore, we recommend that governments and policymakers utilize these platforms to deliver evidence-based information about diseases.

## Data Availability

The datasets used in this study are fully available from the corresponding author upon request.

## Research registration unique identifying number (UIN)

Researchregistry.com (researchregistry10486).
